# ﻿A checklist of Mantodea for Myanmar with the first record of *Schizocephalabicornis* (Mantodea, Eremiaphilidae) for the country

**DOI:** 10.3897/zookeys.1232.141810

**Published:** 2025-03-25

**Authors:** Zohreh Mirzaee, Martin Wiemers, Thomas Schmitt

**Affiliations:** 1 Senckenberg Deutsches Entomologisches Institut, Eberswalder Str. 90, 15374 Müncheberg, Germany Senckenberg Deutsches Entomologisches Institut Müncheberg Germany; 2 Entomology and Biogeography, Institute of Biochemistry and Biology, Faculty of Science, University of Potsdam, D-14476 Potsdam, Germany University of Potsdam Potsdam Germany

**Keywords:** Distribution, mantids, Schizocephalini, taxonomy

## Abstract

This paper presents the first comprehensive checklist of Mantodea species in Myanmar, reporting a total of 54 species belonging to 11 families and 32 genera, one of which (i.e., *Schizocephalabicornis*) constitutes a new record for the country. Four species, *Creobroterurbanus*, *Gonypetabrunneri*, *Theopompaservillei*, and *Rhomboderalaticollis*, are considered doubtful within Myanmar’s fauna and require further investigation to confirm their presence. Additionally, *Aethalochroaashmoliana*, *Gonypetapunctata*, and *Toxoderopsistaurus* are regarded as erroneous records.

## ﻿Introduction

The study of Mantodea in Myanmar has been largely neglected, with most records being scattered across various publications that primarily focus on the Mantodea of other regions (e.g., Borneo, India) or the catalogue of Mantodea of the world (Ehrmann 2002; Schwarz et al. 2018; Sathe and Vaishali 2014; Mukherjee et al. 2014; Ehrmann and Borer 2015; Ghate et al. 2021; Yadav and Painkra 2021; Wu and Liu 2021; Kamila and Sureshan 2022). These studies often mention species that also occur in Myanmar. However, until now, there has been no comprehensive checklist dedicated to the Mantodea of Myanmar. This study closes this gap and presents the first such checklist of Mantodea from Myanmar, by using all the relevant literature in order to provide a comprehensive view of the species present in this region. The creation of a checklist is crucial for several reasons. First, a checklist facilitates taxonomic research by providing a reliable reference that can be used to compare species distributions and identify gaps in current knowledge (Margules and Pressey 2000). Second, it serves as a basic resource for biodiversity assessment and conservation planning, helping to identify species that may be endemic or at risk (Costello et al. 2013). By compiling this checklist, we aim to facilitate and encourage further research on Myanmar’s Mantodea diversity.

## ﻿Materials and methods

To compile the checklist, we utilized a range of literature including Wood-Mason (1889), Ehrmann (2002), Roy (2009), Sureshan and Sambath (2009), Schwarz et al. (2018), Mukherjee et al. (2014), Sathe and Vaishali (2014), Ehrmann and Borer (2015), Yadav and Painkra (2021), Wu and Liu (2021), Kamila and Sureshan 2022, as well as the Mantodea Species File website (Otte et al. 2023). These sources are well established and widely regarded as authoritative references in Mantodea research, frequently cited by experts in the field. In addition to the primary sources, data from the iNaturalist and GBIF platforms were also utilized. The term “Mantodea” was searched using a regional filter set to “Myanmar” to extract relevant records. Since citizen science platforms like GBIF may contain misidentifications, each record, along with its associated photographs when available, was individually reviewed.

The assessments followed specific criteria: If key morphological features could be clearly identified from the photographs, the species record was included in this study. However, if identification required the examination of male genitalia or other characteristics not visible in the photographs, the species was either excluded from the checklist or classified as potentially present. Such records were marked for further verification through future surveys to confirm their presence. This systematic approach improved the reliability of the species records while acknowledging the limitations of photographic evidence in certain cases. The taxonomy employed in this study adheres to the framework proposed by Schwarz and Roy (2019), with classification levels – family, subfamily, tribe, subtribe, genus, and species – structured according to the system detailed by Otte et al. (2023).

For the assignment of faunal elements, we first distinguished species apparently endemic to Myanmar from all the others. Second, we selected the species with a Bengal distribution, i.e., adjoining the Gulf of Bengal, being distributed along the coast of east India, as well as in Bangladesh and Myanmar. For the remaining species, the Oriental realm sensu Wallace (1876) was dissected into three parts: India, Indochina, and Sundaland. While India and Indochina are more or less separated by the Ganges delta, Indochina and Sundaland are separated by the Isthmus of Kra in Thailand (Schmitt 2020). If a species is marginally penetrating one adjoining sub-region, it is not considered part of this subregion; thus, Indochinese species also entering north-eastern India are not considered Indian elements, while Indian elements marginally entering Myanmar are not considered as Indochinese elements. As adjoining regions to the Oriental realm, we consider the two transition regions towards East Palearctic and Australis (i.e., Wallacea) and the two regions East Palearctic and New Guinea. Species occurring beyond these regions are classified as such.

Among the unidentified Mantodea specimens housed at the Senckenberg German Entomological Institute (**SDEI**), one specimen was identified by the first author as *Schizocephalabicornis* (Linné, 1758). This identification was achieved through a detailed examination of morphological characteristics, comparing the specimen with other *S.bicornis* specimens from Sri Lanka and India available in the collection. The method for preparing male genitalia followed Brannoch et al. (2017). The final segments of the male abdomen were dissected under a microscope, with the genitalia separated from the terminalia. The genitalia were then macerated in a 10% KOH solution for 24 h. After maceration, the sample was rinsed in distilled water for 24 h, followed by treatment in 70% ethanol, and finally, placed in glycerin to eliminate any remaining ethanol. The genitalia were photographed and stored in a vial with glycerin drops for further analysis. Photographs were taken using a set-up that included the Stone Master Stack Unit, an Olympus OM-D E-M1 Mark II camera, and Zeiss Luminar lenses (40 mm). The software used included Olympus Capture, Stone Master v. 3.8, Helicon Focus 7 for photo stacking, and ImageJ 1.53t for adding scale bars.

To create the distribution map, occurrence records of *S.bicornis* were gathered from previous studies (Sureshan and Sambath 2009; Sathe and Vaishali 2014; Yadav and Painkra 2021), as well as from the GBIF database (https://doi.org/10.15468/dl.vh8rf8), the iNaturalist portal (https://www.inaturalist.org/taxa/52101-Schizocephala-bicornis), and various museum collections (State Museum of Natural History, Karlsruhe (SMNK); Cleveland Museum of Natural History (CMNH); Lund University Biological Museum—Insect Collections Inventory, Entomological Collections (LUZM); Swedish Museum of Natural History (NHRM); and Royal Ontario Museum—Entomology Collection (ROMT)). All records available in GBIF were initially sourced either from iNaturalist or various museum collections. Upon reviewing iNaturalist records, we examined the associated photographs and excluded a few that were incorrectly identified. Subsequently, we contacted the museums to request photographs and the coordinates of the specimens, allowing us to verify that museum records corresponded to *S.bicornis*. A total of 180 records were obtained and mapped using QGIS v. 3.22.

Abbreviations of the zoological institutes and museums mentioned in this study:

**ANSP**Academy of Natural Sciences, Philadelphia, USA

**CNMS**National Museum, Colombo, Sri Lanka

**DBUC**Dipartimento di Biologia Animale, Università di Catania, Catania, Sicily, Italy

**FRID** Forest Research Institute, Dehra-Dun, India

**HNHM**Hungarian Natural History Museum, Budapest, Hungary

**IEAS**Academia Sinica, Shanghai, China

**IFRI**Indian Forest Research Institute, Dehra Dun, Uttar Pradesh, India

**LNHSM** Lingnan Natural History Survey and Museum, Lingnan University, China

**LSUK** Linnean Society, London, United Kingdom

**MEUU**Museum of Evolution of Uppsala University, Uppsala, Sweden

**MHNG**Muséum d’histoire naturelle, Geneva, Switzerland

**MNHN**Muséum national d’Histoire naturelle, Paris, France

**MRSN** Natural History Museum, Turin, Italy

**MSNG**Museo civico di Storia naturale G. Doria, Genoa, Italy

**NHML** Natural History Museum of Los Angeles, USA

**NHMUK**Natural History Museum, London, Great Britain

**NHMW**Natural History Museum, Vienna, Austria

**NHRS**Naturhistoriska Riksmuseet, Stockholm, Sweden

**OXUM** University Museum, Oxford, Great Britain

**RMNH**Nationaal Natuurhistorisch Museum, Leiden, Netherlands

**SDEI** Senckenberg German Entomological Institute, Müncheberg, Germany

**SEM** Shanghai Entomological Museum, Chinese Academy of Sciences, Shanghai, China

**SMNK**State Museum of Natural History, Karlsruhe, Germany

**UZIU** Universitets Zoologiska Institut, Uppsala, Sweden

**ZMAS** Saint-Petersburg, Zoological Institute of the Russian Academy of Sciences, St. Petersburg, Russia

**ZMB**Museum für Naturkunde der Humboldt-Universität zu Berlin, Berlin, Germany

**ZMUH** Zoological Museum and University, Copenhagen, Denmark

**ZSIC**Zoological Survey of India, Calcutta, India

**ZSM**Zoological State Collection, Munich, Germany

## ﻿Results

### ﻿Checklist of the Mantodea of Myanmar

The checklist presented in this study includes a total of 54 species across 11 families and 32 genera. A review of observational records from iNaturalist and GBIF yielded approximately 102 records for 24 species from iNaturalist and 293 records from 47 species from GBIF. The GBIF data consisted of records from multiple sources, including:

- SMNK Mantid Collection: 236 records
- iNaturalist Research-grade Observations: 19 records
- NMNH Material Samples (USNM): 9 records
- NHMUK (London) Collection Specimens: 8 records
- NMNH Extant Specimen Records (USNM): 7 records
- International Barcode of Life project (iBOL): 4 records
- Cleveland Museum of Natural History: 4 records
- Paleobiology Database: 3 records
- INSDC Sequences: 3 records

In total, these platforms provided records for 47 species, including three extinct species of Mantodea (*Burmantisasiatica* Grimaldi, 2003, *Burmantisburmitica* Grimaldi, 2003, *Burmantiszherikhini* Delclos, Penalver, Arillo, Engel, Nel, Azar & Ross, 2016) discovered in Myanmar from amber fossils. Most of the recorded specimens are housed in museums and are included in the literature used for this study. However, some species were identified from Myanmar in museum collections, mostly at SMNK, Germany. Based on the known distribution of these species, it is plausible to classify the following species as expected taxa for Myanmar:

*Anaxarchagraminea* Stål, 1877

*Hierodulatenuidentata* Saussure, 1869

*Hierodulapistillinota* Wang, Zhou & Zhang, 2020

*Hierodulaconfusa* Vermeersch & Unnahachote, 2020

*Tropidomantisgressitti* Tinkham, 1937

*Tropidomantistenera* (Stål, 1858)

*Creobroterapicalis* Saussure, 1869

*Leptomantellatonkinae* Hebard, 1920

*Acromantisgestri* Giglio-Tos, 1915

Additionally, the following species are regarded as doubtful and require verification:

*Gonypetabrunneri* Giglio-Tos, 1915 (Ehrmann 2002)

*Theopompaservillei* (De Haan, 1842) (Mukherjee et al. 2014; Kamila and Sureshan 2022)

*Rhomboderalaticollis* Burmeister, 1838 (Ehrmann 2002)

*Creobroterurbanus* (Fabricius, 1775) (Ehrmann and Borer 2015; Kamila and Sureshan 2022).

Furthermore, the species *Aethalochroaashmoliana* (https://www.inaturalist.org/taxa/750709-Aethalochroa-ashmoliana/browse_photos?place_id=6992), *Gonypetapunctata* (De Haan, 1842) reported in regional checklists (Mukherjee et al. 2014; Kamila and Sureshan 2022), and *Toxoderopsistaurus* (https://www.inaturalist.org/taxa/750765-Toxoderopsis-taurus/browse_photos?place_id=6992) are considered erroneous in this study. The presence of *Gonypetapunctata* (Evgeny Shcherbakov, pers. comm. Dec. 2024), *Aethalochroaashmoliana*, and *Toxoderopsistaurus* in Myanmar is uncertain due to the lack of confirmed specimens, potential misidentifications, and gaps in verified distribution data. Further fieldwork and examination of museum specimens are recommended to confirm the occurrence of these species in Myanmar. This checklist provides a comprehensive compilation based on current knowledge and a thorough review of the literature, with all relevant records carefully examined.

#### ﻿Order Mantodea Latreille, 1802

**Family Metallyticidae Giglio-Tos, 1917**

**Genus *Metallyticus* Westwood, 1835**

**1. *Metallyticusviolaceus* (Burmeister, 1838)**

*Metalleuticaviolacea* Burmeister, 1838: 527.

= *Metallyticussplendidus* var. Westwood, 1835: 442.

= Metallyticussplendidusvar.purpureus Westwood, 1837: 359. Westwood 1889: 1.

= *Metalleuticavitripennis* Burmeister, 1838: 527.

= *Mantischalybea* Serville, 1839: 202–203.

= *Metalleuticaviolacea* Burmeister, 1838: Charpentier 1841: 287–288. Saussure 1871: 267–268. Borre 1883: 62. Wood-Mason 1889: 1.

= Mantis (Metalleutica) splendida Westwood, 1835: De Haan 1842: 83.

**Type material.** Paratypes ♂ ♀ ZMB.

**Type locality.** Java.

**Distribution.** India (?), Myanmar (Giglio-Tos 1927; Wieland 2008), Malay Peninsula, Sumatra (Singkep Island), Borneo, Java, Davao (S Mindanao, Philippines) (Ehrmann 2002).

**Faunal element.** Oriental.

**Remark.** Historical records of *M.violaceus* from Myanmar and neighboring regions contain certain ambiguities. Giglio-Tos (1927) documented a single specimen from Tavoy, Myanmar, now known as Dawei, a city in southeastern Myanmar. This record is considered reliable, as it aligns well with the known distribution of the species. However, Wieland (2008) reported the existence of two specimens collected in 1836, currently housed in the Musée National d’Histoire Naturelle, Paris. These specimens are labelled as originating from “Mari, Indes orientales,” a term that Wieland found ambiguous. His research indicated that “Mari” could refer to several locations, including sites in Pakistan, Myanmar, and Papua New Guinea. Ultimately, Wieland speculated that the location might be in northern Myanmar or Pakistan. Based on the known distribution of this species, we believe that its occurrence in northern Myanmar is unlikely. Instead, it is more plausible that the term “Mari” refers to a location in Indonesia, where species of this genus are commonly found. Further research and clarification of historical records are necessary to accurately determine the origin of these specimens.

#### ﻿Family Amorphoscelidae Stål, 1877

**Subfamily Amorphoscelinae Stål, 1877**

**Genus *Amorphoscelis* Stål, 1871**

**2. *Amorphoscelis* sp.**

**Remark.** In July 2024, a nymph was spotted and recorded on iNaturalist (https://www.inaturalist.org/observations/233030975) from Shan, Myanmar (21.929'N, 99.840'E). This specimen may belong to *Amorphoscelissingaporana* Giglio-Tos, 1915; however, accurate identification of *Amorphoscelis* species currently relies on examining male genitalia. Consequently, additional research is needed to confirm and verify the presence of this species in Myanmar. Additionally, a specimen of *Amorphoscelis* is housed in the Cleveland Museum of Natural History. It was collected by D. Brzoska from Thaung Dut, Sagaing, Myanmar, in 2013 and identified as *Amorphoscelisborneana* Giglio-Tos, 1914. However, due to the lack of access to these specimens and the need for male genitalia examination for definitive identification, we recommend future surveys to validate the occurrence of one or both species in Myanmar.

#### ﻿Family Nanomantidae Brunner de Wattenwyl, 1893

**Subfamily Tropidomantinae Giglio-Tos, 1915**

**Tribe Tropidomantini Giglio-Tos, 1915**

**Genus *Eomantis* Giglio-Tos, 1915**

**3. *Eomantisguttatipennis* (Stål, 1877)**

*Tropidomantisguttatipennis* Stål, 1877: 51.

= *Eomantis* [*Tropidomantis*] *guttatipennis* Stål, 1877: Giglio-Tos 1915: 47.

**Type material.** Holotype ♂ NHRS, paratype: ♀ NHMW.

**Type locality.** Nepal: Himalaya.

**Distribution.** India, Nepal, Tibet (China), Myanmar (Mukherjee et al. 2014; Schwarz et al. 2018), N Vietnam (Ehrmann and Borer 2015).

**Faunal element.** North Oriental.

#### ﻿Family Gonypetidae Westwood, 1889

**Subfamily Iridopteryginae Giglio-Tos, 1915**

**Tribe Amantini Schwarz & Roy, 2019**

**Genus *Amantis* Giglio-Tos, 1915**

**4. *Amantisaliena* Beier, 1930**

*Amantisaliena* Beier, 1930: 439.

**Type material.** Holotype ♀ NHMUK.

**Type locality.** Myanmar-SW: Tenasserim.

**Distribution.** Myanmar.

**Faunal element.** Endemic in Myanmar.

**5. *Amantisbiroi* Giglio-Tos, 1915**

*Amantisbiroi* Giglio-Tos, 1915: 153.

**Type material.** Holotype ♂ HNHM, paratype ♀ MHNG.

**Type locality.** India E: Martheran 800 m, Carin Cheba.

**Distribution.** India, Myanmar (Mukherjee et al. 2014; Schwarz and Konopik 2014).

**Faunal element.** Indian.

**6. *Amantisbolivarii* Giglio-Tos, 1915**

*Amantisbolivarii* Giglio-Tos, 1915: 153.

**Type material.** Syntypes ♂ MHNG, ♀ MSNG.

**Type localities.** Myanmar-SW: Tenasserim, Nepal: Himalaya-Kurseong.

**Distribution.** India, Nepal, Myanmar, Vietnam (Ehrmann and Borer 2015).

**Faunal element.** North Oriental.

**Remark.**Ehrmann (2002) listed the following type material for the species: a male holotype in MHNG, a female paratype in MSNG, a male paratype in HNHM, and a possible (para)type male in MRSN. However, according to the original description, there are only two syntypes, a male from Kurseong and a female from Tenasserim, with no holotype originally designated. Therefore, there are still only two syntypes in MHNG and MSNG, with no holotypes or paratypes nor lectotypes and paralectotypes being designated at this moment.

**7. *Amantisfuliginosa* (Werner, 1931)**

*Cimantisfuliginosa* Werner, 1931: 1330.

**Type material.** Holotype ♂ NHMUK.

**Type locality.** India: Madras-Anamalai Hills, 700 m a.s.l.

**Distribution.** India, Nepal, Myanmar (Mukherjee et al. 2014).

**Faunal element.** Indian.

**8. *Amantisirina* (Saussure, 1870)**

*Gonypetairina* Saussure, 1870: 244. Saussure 1871: 56–57.

= *Iridopteryx*? [*Gonypeta*] *irina* (Saussure, 1870): Kirby 1904: 223.

**Type material.** Holotype ♂ MHNG.

**Type locality.** Maluku Islands.

**Distribution.** Myanmar, Malay Peninsula, Sumatra, Maluku Islands (Ehrmann 2002).

**Faunal element.** Indochinese and Sundaian.

**9. *Amantisreticulata* (De Haan, 1842)**

Mantis (Oxypilus) reticulata De Haan, 1842: 87. Stål 1860: 313–314. Kirby 1904: 223. Rehn 1912: 122.

= *Iridopteryxinfumata* Bolivar, 1897: 305–306. Rehn 1903: 702.

= *Amantisgestri* Giglio-Tos, 1915: Hebard 1920: 30–31. Giglio-Tos 1927: 171. Beier 1935: 28: Beier 1966: 361 (Syn.?).

**Type material.** Holotype ♂ RMNH.

**Type locality.** Java: Karawang.

**Distribution.** Myanmar, Malay Peninsula, Sumatra, Borneo, Palawan, Java (Ehrmann 2002).

**Faunal element.** Indochinese and Sundaian.

#### ﻿Subfamily Gonypetinae Westwood, 1889

**Tribe Gonypetini Westwood, 1889**

**Subtribe Gonypetina Westwood, 1889**

**Genus *Memantis* Giglio-Tos, 1915**

**10. *Memantisfuliginosa* (Thunberg, 1815)**

*Mantisfuliginosa* Thunberg, 1815: 291–292.

= *Gonypetafemorata* Saussure, 1870: 230. Saussure 1871: 58–59.

= *Humbertiellaconsobrina* Saussure, 1871: 273–274.

= *Gonypeta* [*Mantis*] *fuliginosa* (Thunberg, 1815): Wood-Mason 1891. Kirby 1904: 224.

= *Elaea* [*Humbertiella*] *consobrina* (Saussure, 1871): Kirby 1904: 214.

**Type material.** Holotype ♀ MEUU.

**Type locality.** Sri Lanka.

**Distribution.** India, Sri Lanka, Nepal, Myanmar (Ehrmann and Borer 2015).

**Faunal element.** Indian.

**Remark.** Recent research has revealed inaccuracies in the previously documented information about this species. Kris Anderson conducted a thorough investigation into Thunberg’s publications and uncovered key details. Contrary to earlier reports, the holotype is not housed at ZMAS, but at Uppsala University. Additionally, while the type locality was originally recorded as “India E,” Anderson’s research has clarified that the correct location is actually Sri Lanka. This updated information, verified through personal communication with Kris Anderson (Nov. 2024), corrects the inaccuracies presented in the earlier literature.

#### ﻿Genus *Gimantis* Giglio-Tos, 1915

**11. *Gimantisauthaemon* (Wood-Mason, 1882)**

*Gonypetaauthaemon* Wood-Mason, 1882: 21–27.

= *Iridopteryxmarmorata* Brunner Von Wattenwyl, 1893: 65–66.

**Type material.** Holotype ♀ ZSIC.

**Type locality.** Myanmar-SW: Tenasserim, on the Mergui River, Minthantoung.

**Distribution.** India, Myanmar, Thailand, Malay Peninsula (Ehrmann 2002).

**Faunal element.** Oriental.

#### ﻿Subtribe Humbertiellina Brunner de Wattenwyl, 1893

**Genus *Humbertiella* Saussure, 1869**

**12. *Humbertiellaceylonica* Saussure, 1869**

*Humbertiellaceylonica* Saussure, 1869: 62.

= *Theopompaseptentrionum* Wood-Mason, 1891: 64–66.

= *Humbertiella* [*Theopompa*] *septentrionum* (Wood-Mason, 1891): Kirby 1904: 214.

**Type material.** Holotype ♂ MHNG.

**Type locality.** Sri Lanka.

**Distribution.** Sri Lanka, NE India, Nepal, Myanmar (Schwarz et al. 2018).

**Faunal element.** Bengal.

**13. *Humbertiellaindica* Saussure, 1869**

*Humbertiellaindica* Saussure, 1869: 62.

= *Humbertiellaafricana* Rehn, 1912: 106–108. Giglio-Tos 1927: 66.

**Type material.** Holotype ♀ MHNG.

**Type locality.** India.

**Distribution.** Pakistan, India, Sri Lanka, Nepal, S Myanmar (Ehrmann and Borer 2015).

**Faunal element.** Indian.

#### ﻿Family Rivetinidae Ehrmann & Roy, 2002

**Subfamily Deiphobinae Schwarz & Roy, 2019**

**Tribe Deiphobini Schwarz & Roy, 2019**

**Genus *Deiphobe* Stal, 1877**

**14. *Deiphobemesomelas* (Manuel, 1797)**

*Mantismesomelas* Manuel, 1797: 635–636.

= *Mantis mesomelas* Olivier, 1792, attributio erroris.

= *Mantisconspurcata* Lichtenstein, 1802, partim.

= *Deiphobemesomelas*: Giglio-Tos 1927: 487. Patel and Singh 2016: 41.

= *Deiphobeinfuscata*: Ehrmann and Borer 2015: 230–231, 249, ♂ (India, Nepal, Sri Lanka).

= *Deiphobebrunneri*: Ehrmann and Borer 2015: 230, 248, ♂.

= *Deiphobeincisa*: Werner, 1933: 900–901, ♂. Roonwal and Bhasin 1951: 313, 315 (♂) (type catalogue). Marshall 1975: 316 (♂) (type catalogue).

= Deiphobepropeincisa: Lombardo 1991: 379–380 (♀) (Myanmar-NE). Lombardo 1993: 197–198 (Nepal). Mukherjee et al. 1995: 200, 278, 279–280, 281 (India-NW). Ehrmann 2002: 118, ♂ (India, Nepal?). Mukherjee et al. 2014: 40–41 (India, Myanmar, Nepal). Ehrmann and Borer 2015: 230, partim (India, Nepal, Myanmar?). Patel and Singh 2016: 41 (India, Myanmar, Nepal).

= *Deiphobeyunnanensis* Tinkham, 1937: 561–562, ♀ (China: Yunnan). Wang 1993: 105–106. Hua 2000: 21. Ehrmann 2002: 118 (China). Otte and Spearman 2005: 229. Zhu et al. 2012: 184.

= *Sphendaleinfuscata*: Bolivar 1897: 312 (31), ♂, ♀ (India or.). Bolivar 1899: 809.

= *Sphendalerobusta* Kirby, 1904: 86 (Nepal). Kirby 1904: 269. Marshall 1975: 323 (type catalogue).

**Type material.** Holotype ♀, India, depository unknown.

**Type localities.** India, India-NW, China.

**Distribution.** India, Nepal, Myanmar, SW China (Schwarz et al. 2018).

**Faunal element.** North Oriental.

#### ﻿Family Eremiaphilidae Saussure, 1869

**Subfamily Iridinae Westwood, 1889**

**Tribe Schizocephalini Saussure, 1869**

**Genus *Schizocephala* Serville, 1831**

**15. *Schizocephalabicornis* (Linné, 1758)**

Gryllus (Mantis) bicornis Linné, 1758: 426. Linné 1764: 116.

= *Mantisbicornis* Linné, 1758: Linné 1767: 691.

= *Mantisoculata* Fabricius, 1781: 348. Fabricius 1787: 228. Stoll 1787: 32–33, 43. Olivier 1792: 632. Fabricius 1793: 19. Lichtenstein 1802: 20.

= *Mantisstricta* Manuel, 1797: 641.

= *Schizocephala stricta* Olivier, 1792: Serville 1831: 56, attributio erroris

= *Schizocephalaoculata* Fabricius, 1781: Burmeister 1838: 552.

= Mantis (Schizocephala) oculata Fabricius, 1781: Blanchard 1840: 13. Blanchard 1845: 226–227. Blanchard 1850: 13.

**Type material.** Holotype ♀ UZIU.

**Type locality.** India.

**Material examined.** 1 ♂, Gangaw District Mountain, Dudaw Taung, Myanmar, 600 m. 4.07.1938), SDEI.

**Distribution**. India, Nepal, Sri Lanka, Myanmar (this work, new record deposited in SDEI: Gangaw District Mountain, Dudaw Taung, 600 m, 14 July 1938), Thailand, Vietnam.

**Faunal element.** North Oriental.

**Figure 1.**
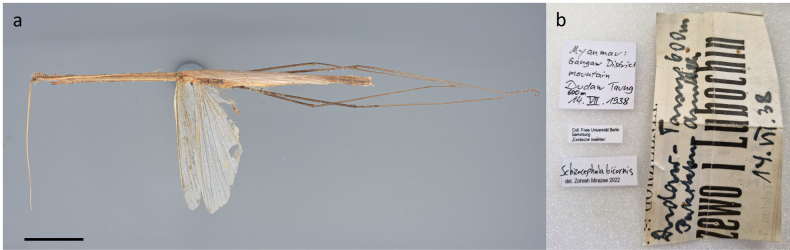
**a***Schizocephalabicornis* male **b** labels. Scale bar: 30 mm (**a**).

**Remark.** This study provides the first occurrence record of *S.bicornis* in Myanmar (Fig. 1). This mantid is characterized by its long and slender body, with coloration ranging from green to pale cream. It has antennae thickened near the base and anteriorly extended eyes that form a cone shape. The fore femur has four posteroventral spines and three discoidal spines, with the second being the longest. The fore tibia is shortened and equipped with six posteroventral spines. In females, the forewing is very small and opaque (Majumder et al. 2015).

#### ﻿Family Toxoderidae Saussure, 1869

**Subfamily Toxoderinae Saussure, 1869**

**Tribe Toxoderini Saussure, 1869**

**Genus *Metatoxodera* Roy, 2009**

**16. *Metatoxoderasubparallela* Roy, 2009**

*Metatoxoderasubparallela* Roy, 2009: 93–183.

**Type material.** Holotype MNHN, paratypes: MNHN (“allotype”), paratypes: NHML, SMNK.

**Type localities.** Myanmar S, Malaysia W.

**Distribution.** Myanmar, Malay Peninsula (Roy 2009).

**Faunal element.** Indochinese and Sundaian.

#### ﻿Genus *Paratoxodera* Wood-Mason, 1889

**17. *Paratoxoderameggitti* Uvarov, 1927**

*Paratoxoderameggitti* Uvarov, 1927: 658–659.

**Type material.** Holotype ♂ NHMUK.

**Type localities.** Burma (Myanmar).

**Distribution.** Myanmar, S China, Malay Peninsula, Borneo (Roy 2009).

**Faunal element.** Indochinese and Sundaian.

#### ﻿Genus *Toxodera* Serville, 1837

**18. *Toxoderabeieri* Roy, 2009**

*Toxoderabeieri* Roy, 2009: 111–117.

**Type material.** Holotype ♂ MHNG, paratypes: ♀ MHNG (“allotype”), ♂ MHNG, ♂ MNHN, ♂ SMNK, ♂ ZSM, ♀ NHML.

**Type localities.** West Malaysia.

**Distribution.** Myanmar, Laos, Malay Peninsula, NW Sumatra, Java, Bali (Roy 2009).

**Faunal element.** Indochinese and Sundaian.

**Remark.** Paratypes have no locality data.

**19. *Toxoderadenticulata* Serville, 1837**

*Toxoderadenticulata* Serville, 1837: 28–29.

= *Toxoderagigas* Ouwens, 1913: 123.

**Type material.** Holotype ♂ MNHN.

**Type locality.** Java.

**Distribution.** India, S China, Myanmar, Thailand, Laos, Malay Peninsula, Sumatra, Borneo, Java (Roy 2009).

**Faunal element.** Oriental.

**20. *Toxoderafimbriata* Werner, 1930**

*Toxoderafimbriata* Werner, 1930: 9.

= *Toxoderaspinigera* Beier, 1931: 20–21.

**Type material.** Holotype ♂ NHRS.

**Type locality.** Sumatra.

**Faunal element.** Indochinese and Sundaian.

**Distribution.** Myanmar, Malay Peninsula, Sumatra, Borneo (Roy 2009).

**21. *Toxoderaintegrifolia* Werner, 1925**

*Toxoderaintegrifolia* Werner, 1925: 485–486.

**Type material.** Holotype ♀ NHRS.

**Type locality.** Java.

**Distribution.** Myanmar, Thailand, Malay Peninsula, Java (Roy 2009).

**Faunal element.** Indochinese and Sundaian.

#### ﻿Family Empusidae Burmeister, 1838

**Subfamily Empusinae Burmeister, 1838**

**Tribe Empusini Burmeister, 1838**

**Subtribe Empusina Burmeister, 1838**

**Genus *Gongylus* Thunberg, 1815**

**22. *Gongylusgongylodes* (Linné, 1758)**

Gryllus (Mantis) gongylodes Linné, 1758: 426. Linné 1764: 112.

= *Mantisgongylodes* Linné, 1758: Linné 1767: 690. Drury 1770: 129–130. Fabricius 1775: 275. Goeze 1778: 22. Fabricius 1781: 346. Fabricius 1787: 227. Gmelin-Linné 1790: 2049. Olivier 1792: 626–627. Fabricius 1793: 17. Lichtenstein 1802: 21–22. Brullé 1835: 78–79.

= *Mantisflabellicornis* Fabricius, 1793: 16–17. Lichtenstein 1802: 22. Latreille 1802: 90. Stoll 1813: 49–50.

= *Empusa* [Gryllus (Mantis)] *gongylodes* (Linné, 1758): Latreille 1807: 90. Stoll 1813: 46–48. Serville 1831: 48. Westwood 1837: 121–122. Serville 1839: 141–142. Charpentier 1841: 296–297.

= *Empusaflabellicornis* (Fabricius, 1793): Serville 1831: 48.

= Empusa (Gongylodes) [Gryllus (Mantis)] *gongylodes* (Linné, 1758): Burmeister 1838: 545.

= Empusa (Empusa) [Gryllus (Mantis)] *gongylodes* (Linné, 1758): Blanchard 1840: 10–11.

= *Gongylus* [Gryllus (Mantis)] *gongyloides* (Linné, 1758): Brunner Von Wattenwyl 1892: 76. Bolivar 1897: 316.

**Type material.** Holotype ♂ UZIU.

**Type locality.** India.

**Distribution.** India, Sri Lanka, Myanmar, Thailand, Java (Schwarz et al. 2018).

**Faunal element.** Oriental.

**23. *Gongylustrachelophyllus* Burmeister, 1838**

Empusa (Gongylus) trachelophylla Burmeister, 1838: 545.

= *Empusa [Gongylus] trachelophylla* Burmeister, 1838: Charpentier 1841: 297.

**Type material.** Type? 2♀♀ ZMB.

**Type locality.** India-E: Bengal, Lamar, Picot.

**Distribution.** India, Myanmar (Wood-Mason 1878).

**Faunal element.** Oriental.

**Remark.** In 1871, Wood-Mason received a specimen from Pegu (now Bago), Myanmar, collected by Mr. S. Kurz during a botanical expedition. He noted that the specimen has only slight differences from the typical form of the species, and its prothoracic shield displayed a striking bright blue-violet coloration. Further investigation is required to verify the presence of this species in Myanmar and determine whether it truly belongs to this species or represents a different one.

#### ﻿Family Hymenopodidae Giglio-Tos, 1915

**Subfamily Hymenopodinae Giglio-Tos, 1915**

**Tribe Anaxarchini Giglio-Tos 1919**

**Genus *Odontomantis* Saussure, 1871**

**24. *Odontomantisplaniceps* (De Haan, 1842)**

Mantis (Oxypilus) planiceps De Haan, 1842: 88.

= Mantis (Oxypilus) planiceps De Haan, 1842: Giebel 1861: 112–113.

= *Acromantisjavana* Giglio-Tos, 1915: Saussure 1870: 230.

= *Odontomantisjavana* (Giglio-Tos, 1915): Saussure 1871: 33. Stål 1877: 87.

**Type material.** Holotype ♂ RMNH, paratypes: ♀ RMNH.

**Type locality.** Holotype: Java (♂), paratype: Borneo (♀).

**Distribution.** Myanmar (this work), Sumatra, Borneo, Java (Ehrmann 2002).

**Faunal element.** Indochinese and Sundaian.

**Remark.** This study documents the presence of this species in Myanmar, i.e., Yangon (16.842°N, 96.174°E), Pyin Oo Lwin (22.039°N, 96.472°E), Ye-U (22.763°N, 95.428°E), based on observations from iNaturalist (https://www.inaturalist.org/observations/139537130, https://www.inaturalist.org/observations/132144778, https://www.inaturalist.org/observations/147323093). However, additional research is required to confirm and validate these records. Additionally, two specimens housed at SMNK in Germany, identified as *Odontomantis* sp. from Myanmar, require further research to determine whether they belong to *Odontomantisplaniceps*.

#### ﻿Tribe Hymenopodini Giglio-Tos, 1915

**Subtribe Hymenopodina Giglio-Tos, 1915**

**Genus *Theopropus* Saussure, 1898**

**25. *Theopropuselegans* (Westwood, 1832)**

*Blephariselegans* Westwood, 1832: 190–191.

= *Creobotra* [*Blepharis*] *elegans* (Westwood, 1832): Saussure 1871: 145. Brunner Von Wattenwyl 1898: 215.

= *Theopropuspraecontatrix* Saussure, 1898: 205 (♀).

= Theopropuselegansvar.flavicans Giglio-Tos, 1927: 562.

= Theopropuselegansvar.rubrobrunneus Beier, 1931: 153.

**Type material.** Holotype ♀ ZSIC.

**Type locality.** Tanesserim coast (Myanmar).

**Distribution.** Myanmar, Malay Peninsula, Sumatra, Borneo, Java (Ehrmann 2002).

**Faunal element.** Indochinese and Sundaian.

#### ﻿Genus *Hymenopus* Audinet-Serville, 1831

**26. *Hymenopuscoronatus* (Olivier, 1792)**

*Mantiscoronata* Olivier, 1792: 638.

= *Mantiscornuta* Olivier, 1792: Lichtenstein 1802: 24–25.

= *Empusabicornis* Stoll, 1787: Latreille 1807: 90.

= *Mantisbicornis* Stoll, 1787: Stoll 1813: 38.

= *Hymenopa* [*Mantis*] *coronata* (Olivier, 1792): Serville 1839: 163.

= *Hymenopus* [*Mantis*] *bicornis* (Stoll, 1787): Saussure 1871: 143. Wood-Mason 1878: 586. Saussure 1898: 209–210. Annandale 1901: 839–848. Shelford 1902: 232. Shelford 1903: 299–304. Pocock 1910: 839. Meade-Waldo 1910: 50–52.

**Type material.** Holotype ♀ ZMB.

**Type locality.** Ambon Island (Amboina), Java.

**Distribution.** NE India, Myanmar (this study), S China, Vietnam, Thailand, Sumatra, Nias, Borneo, Java, Ambon, Flores (Hebard 1920; Werner 1921, 1933; Beier 1942).

**Faunal element.** Indochinese and Sundaian.

**Remark.** This species is documented for the first time in Myanmar through observational records available on iNaturalist (https://www.inaturalist.org/observations/165655863, https://www.inaturalist.org/observations/165655863, https://www.inaturalist.org/observations/143420594) and ten specimens housed in SMNK, Germany.

#### ﻿Subtribe Pseudocreobotrina Brunner de Wattenwyl, 1893

**Genus *Creobroter* Westwood, 1889**

**27. *Creobrotergemmatus* (Houttuyn in Stoll, 1813)**

*Mantisgemmata* Houttuyn in Stoll, 1813: 71.

= *Creobotra* [*Mantis*] *gemmata* (Stoll, 1813): Saussure 1869: 72–73, attributio erroris

= *Creobotra* [*Mantis*] *urbana* (Fabricius, 1775): Saussure 1871: 144–145. Brunner Von WattenwyL 1893: 73. Bolivar 1897: 315.

= *Creoboter* [*Mantis*] *gemmata* (Stoll, 1813): Kirby 1904: 291, attributio erroris

= *Creobrotergemmatus*: Beier 1929: 251–252. Ingrisch 1987: 136 (Nepal). Ehrmann 2002: 112. Otte and Spearman 2005: 89. Zhu et al. 2012: 47–49, ♂ and ♀. Ehrmann and Borer 2015: 230, 247, ♀. Patel et al. 2016c: 42051.

**Type material.** Holotype ♂ (lost).

**Type locality.** Unknown.

**Distribution.** India, Nepal, Myanmar (Ehrmann and Borer 2015; Kamila and Sureshan 2022), Thailand, S China, Vietnam, Sunda Islands (Ehrmann 2002).

**Faunal element.** Oriental.

**Remark.** Regarding the large disjunction and broad distribution of species within the *Creobroter* genus, it is probable that the extensive ranges reported for some species in the literature are artifacts of misidentification. We propose that this may have occurred due to the distributions of several closely related species being mistakenly combined into the range of a single species. The genus *Creobroter* includes 23 described species (Ehrmann 2002; Zhu et al. 2012), most of which exhibit considerable morphological similarity. Notably, *C.gemmatus* has often been used as a “standard identification” for any *Creobroter* specimen that could not be classified into another species, which may have led to subsequent descriptions referencing material originally assigned to *C.gemmatus*. Moreover, the genus has never been comprehensively revised, and many type specimens of early-described species are either lost or irreparably damaged. As a result, the taxonomy of this genus remains problematic until these issues are thoroughly addressed.

#### ﻿Subfamily Phyllothelyinae

**Tribe Parablepharini**

**Genus *Parablepharis* Saussure, 1870**

**28. *Parablephariskuhlii* (De Haan, 1842)**

Mantis (Blepharis) kuhlii De Haan, 1842: 93–94.

**Type material.** Holotype ♀ RMNH.

**Type locality.** Java.

**Distribution.** NE India, Myanmar, Vietnam, Borneo, Java (Ehrmann 2002; Mukherjee et al. 2014; Ehrmann and Borer 2015; Kamila and Sureshan 2022).

**Faunal element.** Indochinese and Sundaian.

#### ﻿Tribe Phyllothelyini Brunner de Wattenwyl 1893

**Genus *Phyllothelys* Wood-Mason, 1877**

**29. *Phyllothelysbreve* (Wang, 1993)**

*Kishinouyeumbreve* Wang, 1993: Ehrmann and Roy 2009: 74.

**Type material.** Holotype ♂ SEM.

**Type locality.** Yunnan, Damenglong.

**Distribution.** Myanmar, China, Laos (Shcherbakov and Anisyutkin 2018; Wu and Liu 2021).

**Faunal element.** Indochinese.

**30. *Phyllothelysparadoxum* Wood-Mason, 1885**

*Phyllothelysparadoxum* Wood-Mason, 1884: 209–210.

**Type material.** Holotype ZSIC.

**Type locality.** Burmah (Myanmar).

**Distribution.** Myanmar.

**Faunal element.** Endemic in Myanmar.

**31. *Phyllothelyswestwoodi* (Wood-Mason, 1876)**

*Phyllocraniawestwoodi* Wood-Mason, 1876: 176. Reprint: 1876: 506–507.

= *Phyllothelis* [*Phyllocrania*] *westwoodii* (Wood-Mason, 1876): Westwood 1889: 44.

**Type material.** Syntypes: ♂ ♀ ZSIC, ♂ NHMUK.

**Type locality.** India: (Assam, Bhutan), Myanmar: Tenasserim.

**Distribution.** NE India, SW Myanmar (Roy 2009).

**Faunal element.** Bengal.

#### ﻿Subfamily Oxypilinae Saussure, 1871

**Tribe Oxypilini Saussure, 1871**

**Genus *Ceratomantis* Wood-Mason, 1876**

**32. *Ceratomantissaussurii* Wood-Mason, 1876**

*Ceratomantissaussurii* Wood-Mason, 1876: 175. Reprint: 1876: 506–507.

= *Oxypilus* [*Ceratomantis*] *saussurii* (Wood-Mason, 1876): Wood-Mason 1879: 259.

**Type material.** Holotype ♂ ZSIC.

**Type locality.** Pegu (Myanmar).

**Distribution.** Myanmar, Thailand, S China, Laos, Malay Peninsula (Ehrmann 2002).

**Faunal element.** Indochinese and Sundaian.

#### ﻿Tribe Hestiasulini Giglio-Tos, 1915

**Genus *Catestiasula* Giglio-Tos, 1915**

**33. *Catestiasulanitida* (Brunner, 1893)**

*Pachymantisnitida* Brunner von Wattenwyl, 1892: 72–73.

= *Catestiasulanitidae* Brunner von Wattenwyl, 1892: Giglio-Tos 1915: 101. Giglio-Tos 1927: 547.

= *Catestiasulanitida* (Brunner von Wattenwyl, 1892): Beier 1958: 247.

**Type material.** Holotype ♂ NHMW.

**Type localities.** Myanmar-SW: Tenasserim near Mount Mooleyit, 1800–1900 m.

**Distribution.** S Myanmar, Malay Peninsula, Sumatra, Borneo, Java (Ehrmann 2002).

**Faunal element.** Indochinese and Sundaian.

#### ﻿Subfamily Acromantinae Brunner de Wattenwyl, 1893

**Tribe Acromantini Brunner de Wattenwyl, 1893**

**Genus *Ambivia* Stål, 1877**

**34. *Ambiviapopa* Stål, 1877**

*Ambiviapopa* Stål, 1877: 88. Westwood 1889: 22, 26.

= *Popa* [*Mantis*] *undata* (Fabricius, 1793): Rehn 1903: 718. Werner 1908: 123–124. Rehn 1911: 25–26.

= *Ambiviapopa* Stål, 1877: Giglio-Tos 1914: 86. Giglio-Tos 1915: 8. Werner 1922: 125. Giglio-Tos 1927: 529–530. Uvarov 1927: 90. Rehn 1927: 51–52. Sjöstedt 1930: 13. Werner 1933: 901. Beier 1956: 40. Mukherjee and Hazra 1982: 464. Lombardo 1993: 204. Mukherjee and Hazra 1993: 497, 500, 506. Lombardo 1995: 258–260. Mukherjee et al. 1995: 212–213.

**Type material.** Holotype ♂ NHRS.

**Type locality.** India: Tranquebar (Kalkutta).

**Distribution.** India, Nepal, Sri Lanka, Myanmar, Thailand, Laos, Vietnam, Malay Peninsula, Sumatra, Borneo (Schwarz et al. 2018).

**Faunal element.** Oriental.

#### ﻿Genus *Acromantis* Saussure, 1870

**35. *Acromantisindica* Giglio-Tos, 1915**

*Acromantisindica* Giglio-Tos, 1915: 7.

**Type material.** Holotype ♀ ZSIC.

**Type locality.** Myanmar: Thngannyinaung, Myavadi.

**Distribution.** S Myanmar (Ehrmann 2002).

**Faunal element.** Endemic in Myanmar.

#### ﻿Family Deroplatyidae Westwood, 1889

**Subfamily Deroplatyinae Westwood, 1889**

**Tribe Deroplatyini Westwood, 1889**

**Subtribe Pseudempusina Rehn, 1911**

**Genus *Pseudempusa* Brunner von Wattenwyl, 1893**

**36. *Pseudempusapavonina* Giglio-Tos, 1916**

*Pseudempusapavonina* Giglio-Tos, 1916: 3.

**Type material.** Holotype ♀ MSNG?

**Type locality.** Myanmar-NE: Carin Chebà, 900–1100 m.

**Distribution.** N Myanmar, N Thailand (Ehrmann 2002).

**Faunal element.** Indochinese.

**Remark.** The holotype might be lost.

**37. *Pseudempusapinnapavonis* Brunner von Wattenwyl, 1892**

*Pseudempusapinnapavonis* Brunner von Wattenwyl, 1892: 75.

**Type material.** Holotype ♀ MSNG.

**Type locality.** Myanmar-NE: Mount Catcin: east of the city, Bhamo, Myanmar: Carin Chebà. 900–1100 m.

**Distribution.** India, Myanmar, Thailand (Ehrmann 2002).

**Faunal element.** North Oriental.

#### ﻿Subtribe Deroplatyina Westwood, 1889

**Genus *Deroplatys* Westwood, 1839**

**38. *Deroplatysangustata* Westwood, 1841**

*Deroplatysangustata* Westwood, 1841: 34.

= *Deroplatyshorrifica* Westwood, 1889: 40. Kirby 1904: 282.

**Type material.** Holotype ♂ OXUM.

**Type locality.** Java.

**Distribution.** Myanmar, Malay Peninsula, Sumatra, Borneo, Java (Ehrmann 2002).

**Faunal element.** Indochinese and Sundaian.

**39. *Deroplatystrigonodera* Westwood, 1889**

*Deroplatystrigonodera* Westwood, 1889: 40.

**Type material.** Holotype ♀ OXUM.

**Type locality.** Burmah (Myanmar).

**Distribution.** Myanmar, Sumatra, Borneo (Ehrmann 2002).

**Faunal element.** Indochinese and Sundaian.

#### ﻿Family Mantidae Latreille, 1802

**Subfamily Choeradodinae Saussure, 1869**

**Genus *Asiadodis* Roy, 2004**

**40. *Asiadodisyunnanensis* (Wang & Liang, 1995)**

*Choeradodisyunnanensis* Wang & Liang, 1995: 84.

**Type material.** Holotype ♂ IEAS.

**Type locality.** Yunnan.

**Distribution.** S China, Myanmar, N Thailand (Roy 2004).

**Faunal element.** Indochinese.

**Remark.** In Asia, *Asiadodissquilla* is broadly distributed across central and southern India as well as Sri Lanka, whereas *A.yunnanensis* is found in southern China, Myanmar, and northern Thailand. However, these areas have not been fully surveyed (Roy 2004).

#### ﻿Subfamily Mantinae Latreille, 1802

**Genus *Mantis* Linné, 1758**

**41. *Mantisreligiosa* Linné, 1758**

Gryllus (Mantis) religiosus Linné, 1758: 426. Scopoli 1763: 105. Seba 1765, 4: 29, 75. Linné 1767: 690.

= *Mantisoratoria* Fabricius, 1775: 276–277. Lichtenstein 1802: 28–29.

= *Mantissancta* Fabricius, 1787: 228. Olivier 1792: 628–629.

= Mantisreligiosavar.striata Fabricius, 1793: 20.

= *Mantismaroccana* Thunberg, 1815: 287–299.

= *Mantis piа* Audinet-Serville, 1839: 193. Kirby 1899: 348.

= *Mantisprasina* Audinet-Serville, 1839: 195. Stål 1877: 61.

= *Mantisradiata* Motchoulsky: Fischer-Waldheim 1846: 101.

= *Mantiscapensis* Saussure, 1872: 46–47. Stål 1877: 60–61.

= Mantisreligiosavar.major Gerstaecker, 1873: 12.

= *Mantismacroura*: Brunner de Wattenwyl 1882: 60.

= *Mantiscarinata* Cosmovici, 1888: 172–173.

= Mantisreligiosaab.flava Padewieth, 1900: 20.

= Mantisreligiosaab.brunnea Padewieth, 1900: 20.

**Type material.** Holotype ♂, paratypes 3 ♀ LSUK.

**Type locality.** Africa.

**Distribution.** Africa, Asia, Europe, North America (introduced) (Ehrmann 2002).

**Faunal element.** Old world.

**Remark.** Originally found in Africa, Europe, and Asia, it has also been introduced to North America.

#### ﻿Genus *Statilia* Stål, 1877

**42. *Statilia* sp.**

**Remark.** In the present study, a specimen from Myanmar is documented as *Statilia* sp. (potentially *Statiliamaculata*), based on an observation from iNaturalist (https://www.inaturalist.org/observations/198274625). Additionally, five specimens identified as *Statilia* and collected from Myanmar are housed at SMNK in Germany, requiring further research for species-level identification. The preliminary identification of *Statilia* sp. by the first author remains provisional, as accurate identification of this genus currently necessitates the examination of male genitalia.

**43. *Statilianobilis* (Brunner von Wattenwyl, 1893)**

*Mantisnobilis* Brunner von Wattenwyl, 1893: 70. Beier 1935: 92. Beier 1942: 142. Mathur 1946: 101. Roonwal and Bhasin 1951: 317. Roy 1967: 127, 148. Roy 1968: 175 (syn. of *Statilianemoralis*).

= *Statilianemoralis*: Chatterjee and Mukherjee 2013: 4907–4909. Ehrmann and Borer 2015: 242, 268. Otte and Spearman 2005: 193. Patel and Singh 2016: 31. Ingrisch 1987: 114, 136, L3 = ♂ (Nepal). Chatterjee and Mukherjee 2013: 4907–4909.

= *Mantisindica* Mukherjee, 1995: 185, 201, 300–301, 357. Roy 2000: 163. Mukherjee and Shishodia 2000: 64, 65. Ehrmann 2002: 215. Mukherjee et al. 2005: 147, #35–36 (type catalog). Otte and Spearman 2005: 185. Vyjayandi 2007: 95. Berg et al. 2011: 44. Ghate et al. 2012: 22. Chatterjee and Mukherjee 2013: 4907–4909. Mukherjee et al. 2014: 3, 38–39. Ehrmann and Borer 2015: 242, 268. Schwarz et al. 2017: 7.

**Type material.** Holotype ♀ MSNG.

**Type locality.** Myanmar; Synonym: *S.indica*: holotype and paratype: India-NE.

**Distribution.** India, Nepal, Myanmar, Thailand (Ehrmann 2002; Kamila and Sureshan 2022).

**Faunal element.** North Oriental.

#### ﻿Subfamily Tenoderinae Brunner de Wattenwyl, 1893

**Tribe Tenoderini Brunner de Wattenwyl, 1893**

**Subtribe Tenoderina Brunner de Wattenwyl, 1893**

**Genus *Tenodera* Burmeister, 1838**

**44. *Tenoderaaridifolia* (Houttuyn in Stoll, 1813)**

*Mantisaridifolia* Houttuyn in Stoll, 1813: 65–66. Audinet-Serville 1839: 178–179, ♂.

= *Paratenoderaaridifolia*: Rehn 1903: 705. Rehn 1909: 180.

= *Tenoderaaridifolia*: Giglio-Tos 1927: 414, ♂ and ♀ (Asia orientalis). Ehrmann 2002: 349. Ehrmann 2002: 349. Zhu et al. 2012: 221, ♂. Schwarz and Konopik 2014: 151. Ehrmann and Borer 2015: 242, 268, ♀. Patel and Singh 2016a: 37. Mukherjee et al. 2017: 9835–9836, ♂.

= *Tenoderaaridifoliaaridifolia*: Beier 1935: 93. Ingrisch 1987: 114, 136, ♂ and ♀ (Nepal). Otte and Spearman 2005: 200.

= *Mantischloreudeta* Burmeister, 1838: 535 (Java, East India). Saussure 1869: 69. Giglio-Tos 1912: 37.

**Type material.** Holotype ♂ ZMUH.

**Type locality.** India-E.

**Distribution.** India, Nepal, S China, Taiwan, Japan, Myanmar, Thailand, Malay Peninsula (Penang, Perak), Philippines, Sumatra, Borneo, Sulawesi, Java, Lesser Sunda Islands (Lombok, Flores, Sumba); introduced: Florida (USA) (Ehrmann 2002; Mukherjee et al. 2014; Ehrmann and Borer 2015; Schwarz et al. 2018; Kamila and Sureshan 2022).

**Faunal element.** Oriental; Wallacean.

**45. *Tenoderafasciata* (Manuel, 1787)**

*Mantisfasciata* Manuel, 1787: 640 (Surinam) (err.).

= *Thespisfasciata*: Audinet-Serville 1831: 46, 55 (Surinam) (err.).

= Mantis (Tenodera) fasciata: Burmeister 1838: 534–535 (Amboina, Java, Tranquebar).

= *Tenoderafasciata*: Giglio-Tos 1912: 45–46. Giglio-Tos 1927: 416. Ingrisch 1987: 114, 136, ♂ (Nepal). Ehrmann 2002: 350. Schwarz and Konopik 2014: 151. Ehrmann and Borer 2015: 243, 269. Patel and Singh 2016a: 38.

= *Tenoderafasciatafasciata*: Otte and Spearman 2005: 202.

= *Mesopteryxfasciata*: Kirby 1904: 238.

= *Mantisleptelytra* Lichtenstein, 1802: 20 (Surinam) (err.). Balderson 1984: 11 (type catalog).

= *Mantisattenuata* Stoll, 1813: 13♂ (Surinam) (err.).

= *Tenoderaattenuata*: Hebard 1920: 51, ♂ (Java). Werner 1922: 152. Balderson 1984: 11 (type catalog)

= *Mantisexsiccata* Audinet-Serville, 1839: 176 (Java). Balderson 1984: 11 (type catalog)

= *Tenoderaintermedia* Saussure, 1870: 233. Balderson 1984: 11 (type catalog)

= *Tenoderasuperstitiosa*: Saussure 1871a: 99. Westwood 1889: 13. Rehn 1903: 705. Werner 1908: 118. Rehn 1909: 180.

**Type material.** See remark.

**Type locality.** Unknown.

**Distribution.** India, Sri Lanka, Nepal, S China, Myanmar, Thailand, Malay Peninsula, Borneo, Sulawesi, Java, Flores, Sumba, Moluccas (Ehrmann 2002; Mukherjee et al. 2014; Ehrmann and Borer 2015; Schwarz et al. 2018; Kamila and Sureshan 2022).

**Faunal element.** Oriental; Wallacean.

**Remark.** The holotype male of ‘*M.leptelytra*’ (erroneously reported from Surinam) is housed in the MNHN, with the depository of the holotype male (*M.fasciata*) remaining unknown. ‘*Mantisattenuata*’ is represented by a holotype male and paratype female from Indonesia (Maluku Islands), with their depository also unknown. Similarly, the holotype female of ‘*M. exsiccataI*’ is from Java, possibly held at MIZT, while *T.intermedia*, based on a holotype female from New Zealand (erroneously reported), also lacks a known depository.

#### ﻿Genus *Mesopteryx* Saussure, 1870

**46. *Mesopteryxplatycephala* (Stål, 1877)**

= *Tenoderaplatycephala* Stål, 1877: 56. Brunner de Wattenwyl 1893: 67, ♀ and ♂ (Myanmar). Sjöstedt 1930: 10, 42, ♀ (type catalog).

= *Mesopteryxplatycephala*: Wood-Mason 1882: 34–35, ♂ and ♀. Westwood 1889: 14. Kirby 1904b: 237 (India-N, Myanmar). Giglio-Tos 1912: 55–56, ♂and ♀ (Myanmar). Giglio-Tos 1927: 420 (India-E, Myanmar). Beier 1935: 94 (India, Myanmar). Ehrmann 2002: 220, ♀ (Cambodia, India, Myanmar). Ehrmann 2002: 220, ♀ (Cambodia, India, Myanmar). Otte and Spearman 2005: 188, ♀ (Indomalaya). Zhu et al. 2012: 214–215, 1–8, ♀. Mukherjee et al. 2014: 34, ♂ and ♀ (Cambodia, India, Myanmar, Nepal). Ehrmann and Borer 2015: 238, 265, ♂. Patel and Singh 2016: 30.

**Type material.** Holotype female, NHRS.

**Type locality.** Unknown.

**Distribution.** NE India, Nepal, Myanmar, Cambodia (Ehrmann 2002; Mukherjee et al. 2014; Ehrmann and Borer 2015; Schwarz et al. 2018; Kamila and Sureshan 2022).

**Faunal element.** Indochinese.

#### ﻿Subfamily Hierodulinae Brunner von Wattenwyl, 1893

**Tribe Hierodulini Brunner de Wattenwyl, 1893**

**Genus *Rhombomantis* Ehrmann & Borer, 2015**

**47. *Rhombomantistectiformis* (Saussure, 1870)**

*Rhomboderatectiformis* Saussure, 1870: 232–233.

**Type material.** Holotype ♀ MHNG.

**Type locality.** India: Bombay.

**Distribution.** Pakistan, India, Nepal, Myanmar (Schwarz et al. 2018; Kamila and Sureshan 2022).

**Faunal element.** Indian.

**48. *Rhombomantisfusca* (Lombardo, 1992)**

*Rhomboderafusca* Lombardo, 1992: 97–100. Ehrmann 2002: 307. Otte and Spearman 2005: 268. Koçak and Kemal 2008: 46.

= *Hierodulabrachynota*Wang and Dong 1993: 205, 207.

= *Rhomboderabrachynota*: Ehrmann 2002: 306. Otte and Spearman 2005: 267.

= *Hierodulabrachynota*: Koçak and Kemal 2008: 46. Zhu Xiao-Yu. et al. 2012: 246.

**Type material.** Holotype ♂, paratype ♀ Thailand-N: Prov. Chiang Mai-Samoeng, Maetaeng, DBUC.

**Type locality.** Thailand-N: Prov Chiang Mai: Samoeng, Maetaeng.

**Distribution.** China (Yunnan), Myanmar, Thailand, Laos, Malay Peninsula (Ehrmann and Borer 2015; Liu et al. 2021).

**Faunal element.** Indochinese.

#### ﻿Genus *Hierodula* Burmeister, 1838

**49. *Hierodulabhamoana* Giglio-Tos, 1912**

*Hierodulabhamoana* Giglio-Tos, 1912: 92–93.

= *Hierodula* [*Mantis*] *simulacrum* (Fabricius, 1793): Brunner von Wattenwyl 1893: 68.

**Type material.** Holotype ♀ MSNG.

**Type locality.** Myanmar-N: Bhamò.

**Distribution.** Myanmar (Ehrmann 2002).

**Faunal element.** Endemic in Myanmar.

**50. *Hierodulagrandis* Saussure, 1870**

*Hierodulagrandis* Saussure, 1870: 233.

**Type material.** Holotype ♂ MHNG, paratype ♀ MHNG.

**Type locality.** Bangladesh: Sylhit.

**Distribution.** NE India, Bangladesh, Myanmar, China (Ehrmann 2002; Mukherjee et al. 2014; Wang et al. 2020; Kamila and Sureshan 2022).

**Faunal element.** Bengal.

**51. *Hierodulalatipennis* Brunner von Wattenwyl, 1893**

*Hierodulalatipennis* Brunner von Wattenwyl, 1892: 69.

= *Hierodulaunimaculata* nec Olivier, 1792: Zhu et al. 2012: 249–251.

= *Hierodulalatipennis* Brunner von Wattenwyl, 1893: 69. Holotype (♀): Myanmar (Burma),

Carin Ghecù, MSNG.

= *Hierodulamacrodentata* Wang, Zhou & Zhang, 2020: 6. Holotype (♂): China, Yunnan, SEM.

**Type material.** Holotype ♀ MSNG.

**Type locality.** Myanmar: Carin Ghecù, 1300–1400 m.

**Distribution.** China (Yunnan), Myanmar (Ehrmann 2002).

**Faunal element.** Indochinese.

**52. *Hierodulamultispinulosa* Brunner von Wattenwyl, 1893**

*Hierodulamultispinulosa* Brunner von Wattenwyl, 1893: 68–69.

**Type material.** Holotype ♀ NHMW?

**Type locality.** Myanmar-C: Mandalay.

**Distribution.** Myanmar (Ehrmann 2002).

**Faunal element.** Endemic in Myanmar.

**Remark.** The holotype might be lost.

**53. *Hierodulapatellifera* (Audinet-Serville, 1838)**

*Mantispatellifera* Audinet-Serville, 1838: 185–186.

= Mantis (Mantis) patellifera: De Haan 1842: 70.

= *Hierodulapatellifera*: Westwood 1889: 12, 27, 35.

= Hierodula (Hierodula) patellifera: Beier 1935: 203: 83.

= *Hierodulapatelliferapatellifera*: Ehrmann 2002: 180. Chatterjee and Srinivasan 2013: 131–135.

= *Mantisbipapilla* Audinet-Serville, 1839: 188–189 (synonymized by Hebard 1920: 58–59). Charpentier 1841: 291–292.

= Mantis (Mantis) bipapilla: De Haan 1842: 70.

= *Hierodulabipapilla*: Saussure 1871: 79–80. Borre 1883: 68. Westwood 1889: 12, 27, 34–35. Kirby 1904: 245. Shiraki 1911: 324–326. Giglio-Tos 1912: 96–98.

= Hierodula (Hierodula) bipapilla: Giglio-Tos 1927: 448. Vyjayandi and Narendran 2003: 315, 317.

= *Hierodulasimulacrum*: Saussure 1869: 68. Saussure 1871: 77–78. Wood-Mason 1882: 30.

= *Hierodulasimulacrum*: Borre 1883: 68.

= *Hierodulamanillensis* Saussure, 1870: 233 (synonymized by Hebard 1920: 58–59). Ehrmann 2002: 180.

= Hierodula (H.) manillensis: Giglio-Tos 1912: 95–96. Giglio-Tos 1927: 447–448.

= *Hierodularaptoria* Stål, 1877: 38 (female) (synonymized by Giglio-Tos 1927: 447–448).

= *Hieroduladispar* Kirby, 1900: 146–147 (synonymized by Giglio-Tos 1927: 448).

= Hierodula (H.) manillana Giglio-Tos, 1912: 96 (synonymized by Hebard 1920: 58–49). Werner 1926: 228–229. Giglio-Tos 1927: 448. Ehrmann 2002: 180.

= Hierodula (H.) patellifera manillana: Beier 1935: 83.

= *Hierodulapatelliferamanillana*? Giglio-Tos, 1912: Ehrmann 2002: 180.

= *Hierodulasaussurei* Kirby, 1904: 245 (synonymized by Hebard 1920). Werner 1926: 228–229.

= Hierodula (H.) saussurei: Giglio-Tos 1912: 94–95. Giglio-Tos 1927: 447.

= *Hierodulasaussurei*: Werner 1930: 4. Ehrmann 2002: 180.

= Hierodula (H.) assamensisMukherjee et al. 1995: 185, 201, 290–291.

**Type material.** Holotype ♂ MNHN, paratype ♀ MNHN.

**Type locality.** Java.

**Distribution.** India, China-S, Korea, Japan, Taiwan, Myanmar (this study), Philippines, Java, Sumba, New Guinea; introduced: Hawaii (Big Island; Ehrmann 2002).

**Faunal element.** Oriental; Wallacean; New Guinean; East Palearctic.

**Remark.** This species is documented for the first time in Myanmar through observational records available on iNaturalist (https://www.inaturalist.org/observations/142558577, https://www.inaturalist.org/observations/250140183) and two specimens collected from Myanmar housed in Natural History Museum (London).

**54. *Hierodulavenosa* Manuel, 1797**

*Mantisvenosa* Manuel, 1797: 639.

= *Mantisconspurcata* Lichtenstein, 1796: 79–80.

= *Mantispunctata* Stoll, 1813: 49.

= *Mantisvitrea* Stoll, 1813: 15.

= Mantis (Hierodula) hybrida Burmeister, 1838: 536.

= *Mantisbankae* Giebel, 1861: 111.

= *Mantissimilis* Giebel, 1861: 112.

= *Hierodulanovemdentata* Saussure, 1869: 68.

= *Hieroduladaphne* Stål, 1877: 38.

= *Hierodulaathene* Rehn, 1909: 180–182.

**Type material.** Type? ♀ ZMB.

**Type locality.** India-E: Tranquebar.

**Distribution.** India, Myanmar, Philippines, Sumatra, Borneo, Java.

**Faunal element.** Oriental.

## ﻿Discussion

Of the 54 species assigned to a faunal element, only five were endemics of Myanmar and three had a typical Bengalese distribution, one of these also occurring in the East Palearctic. The most common pattern was the combination of an Indochinese and Sundaian distribution (15 species), while pure Indian and pure Indochinese distribution patterns with five and six species, respectively, were relatively rare. The same applies to six species for the combination of Indian and Indochinese distributions (i.e., North Oriental). While all these species had few cases of distributions beyond the Oriental realm, this was commonly observed for the 12 species widely distributed across the three major regions of the Oriental realm (Table 1).

**Table 1.** Numbers of mantid species endemic to Myanmar or present in four distinguished sub-regions of the Oriental realm and their combinations.

Faunal Element	Number of Species
Endemic in Myanmar	5
Bengal	3
Indian	5
Indochinese	6
Indochinese & Sundaian	15
North Oriental (Indian & Indochinese)	6
Oriental (Widespread in Oriental Realm)	12
Oriental & Wallacean	3
Oriental & East Palearctic	5
Old World	1

The checklist provided here aims to build a foundation for future research, offering a comprehensive overview of currently known mantid species in Myanmar. It may facilitate comparative studies with neighboring regions, potentially revealing patterns of species distribution and endemism. It may also help to identify areas where further research is needed, guiding future survey efforts.

The data compiled here, for example, allow a biogeographic assessment of Myanmar and beyond. Thus, the entire mantid fauna of Myanmar clearly belongs to the Oriental realm sensu Wallace (1876). No Palearctic elements enter the country and, considering mantids, also its northernmost parts are clearly Oriental with no Palearctic influences. Interestingly, in the majority of cases the species found in Myanmar are restricted to the Oriental realm. The species passing the Wallace line, hereby entering the transition zone of Wallacea, is limited to three, supporting the general relevance of this biogeographic border (Mayr 1944; Simpson 1977). Only one species advances to New Guinea and hence to Australis, supporting the eastern border of Wallacea (i.e., the Lydekker line) as another important biogeographical border. These findings offer more support to the “old” biogeographical classifications (Wallace 1876; Müller 1980) and align with the multi-taxa study by Holt et al. (2013) classifying southern China as part of the Oriental realm and not as transition zone; these authors did not verify the Wallace line but only the Lydekker line.

Looking at the biogeographic structuring of these mantids within the Oriental realm reveals a rate of close to 10% Myanmar endemics. This is relatively low compared with islands (Kier et al. 2009), but is noteworthy in a country not really geographically isolated from the adjoining areas. This finding also underlies the Myanmar faunal element erected on analyses of distribution data of odonates (Heiser and Schmitt 2013). The more widespread species clearly assigned Myanmar to the Indochinese and not to the Indian sub-region (20 vs 5 species). Interestingly, elements widespread in both these sub-regions (i.e., 6 species) are relatively few, underlining the assumed split between these two regions (Schmitt 2020), in the case of mantids maybe fostered by the Ganges delta. On the contrary, a common distribution in Indochina and Sundaland was observed for as many as 15 mantid species. Thus, the Isthmus of Kra, which has been demonstrated as an important biogeographic breaking-point in many species, including mammals, birds, and plants (Schmitt 2020), apparently is not of major importance for the distribution pattern of mantids. This might be explained by the drying of the sea during glacial periods and the complete exposure of the shelf area, which first was flooded only ca 500 ky ago (Salles et al. 2021). Apparently, mantids had good dispersal conditions via the dried Sunda shelf so that the recent geographic impediment via the Isthmus of Kra seems to be mostly irrelevant for their extant distributions. How this pattern might be reflected in the phylogeographic patterns within species is still awaiting resolution in future research. With a total of 12 species, taxa widely distributed all over the Oriental realm are only a small proportion of all species, thus again underlining the known strong sub-structuring within this realm (Heiser and Schmitt 2013; Schmitt 2020), which also holds true for mantids.

Based on known distributions and ecological factors, several mantid species are expected to occur in Myanmar, although their presence has yet to be confirmed. Species such as *Anaxarchagraminea*, *Hierodulatenuidentata*, *Tropidomantisgressitti*, and *Leptomantellatonkinae* are likely to inhabit the country’s diverse ecosystems, given their presence in neighboring regions with similar environmental conditions. Recent discoveries like *Hierodulapistillinota* and *Hierodulaconfusa* further support the potential for new records with continued research, particularly in underexplored areas. However, some previously reported species, such as *Gonypetabrunneri*, *Theopompaservillei*, and *Rhomboderalaticollis*, remain doubtful due to uncertainties in identification and the lack of confirmed specimens. Similarly, records of *Aethalochroaashmoliana*, *Gonypetapunctata*, and *Toxoderopsistaurus* require further verification, as they may be the result of misidentifications or gaps in distribution data. The use of multiple data sources, including literature, museum collections, and online databases like GBIF and iNaturalist, provides a comprehensive approach to biodiversity documentation. This methodology enhances the reliability of findings and highlights the importance of integrating traditional taxonomic work (Marques et al. 2024). While online platforms offer valuable data, rigorous field studies and examination of museum specimens remain essential to confirm expected species and reassess doubtful records.

We recommend field studies to confirm the presence and evaluate the population status of the species listed in the checklist. Incorporating genetic studies might provide deeper insights into the biogeography of species, on the one hand maybe uncovering cryptic diversity and differentiation, or, on the other hand, potentially confirming population connectivity across entire ranges. Given the evolving nature of taxonomic research and the continuous emergence of new discoveries, regular updates to the checklist are crucial to maintain its relevance and accuracy (Marques et al. 2024).

**Figure 2.**
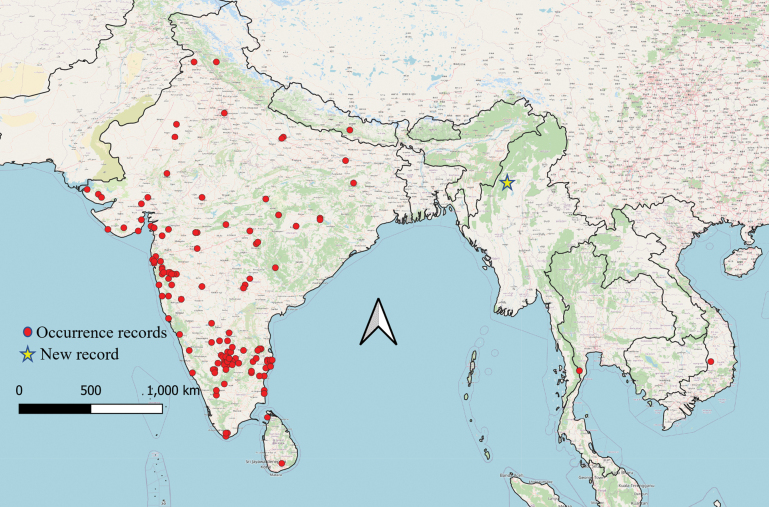
Distribution records of *Schizocephalabicornis*. Red circles representing previously documented locations, while the blue star highlights the newly identified record from Myanmar reported in this study and partly closing the formerly existing gap in the distribution.

One highlight of our checklist is the discovery of *Schizocephalabicornis* (Linné, 1758) in Myanmar expanding our understanding of the species’ distribution in south-eastern Asia. This species is commonly known as the Indian grass mantid, as it is widespread particularly in southern India. It is a large species, with females up to 14.5 cm in length (Yadav and Painkra 2021) and belongs to the monotypic tribe Schizocephalini (Schwarz and Roy 2019). This mantis is a long, slender species that mimics grass, characterized by a narrow head with forward-protruding conical eyes, a triangular metazona, elongated slender legs, shortened fore coxae, and a long, triangular supra-anal plate (Fig. 1; Majumder et al. 2015). Its natural habitat consists of wet savannahs and high-growing grass layers, in which individuals can optimally camouflage themselves (Mukherjee et al. 1995). Although the presence of *S.bicornis* in Myanmar is not unexpected, given its known distribution in neighboring countries (Fig. 2; Mukherjee et al. 2017), this new record fills the hitherto assumed distribution gap, suggesting a more continuous distribution across the Indian subcontinent and south-eastern Asia. This discovery also underlines the need for more surveys and biodiversity assessments in this region to better understand the extant distributions of Mantodea species.

In summary, this study not only broadens our understanding of Mantodea distribution in south-eastern Asia but also serves as a valuable resource for future entomological research in Myanmar. The combination of new distribution records and a comprehensive species checklist lays the basis for more detailed studies of Myanmar’s rich insect fauna, thereby contributing to broader efforts in biodiversity conservation and taxonomic research in this region.

## ﻿Additional information

### Author ORCIDs

Zohreh Mirzaee https://orcid.org/0000-0002-1496-8253

Martin Wiemers https://orcid.org/0000-0001-5272-3903

Thomas Schmitt https://orcid.org/0000-0002-1389-8396

### Data availability

All of the data that support the findings of this study are available in the main text.
